# Using Metrics to Improve Population Health

**Published:** 2010-06-15

**Authors:** Robert M Pestronk

**Affiliations:** National Association of County and City Health Officials

## Introduction

The Mobilizing Action for Community Health (MATCH) project proposes an incentive system that would reward improved health at the population level. Such incentives depend on metrics, but how should metrics be selected?

A logic model with theoretical, philosophical, or political grounding is an essential first step. A model conceptualizes the production of population health, and metrics are chosen on the basis of that conceptualization. To achieve population health, for example, should we seek improvements in access to care, in medical or disease conditions, or in the social, political, and economic underpinnings of society itself? Metrics are the yardstick by which assumptions in the model will be tested. They measure evidence of actual inputs, outputs, and outcomes. When choosing metrics associated with incentives, we must decide what type or magnitude of change we seek.

What population's health should improve? Metrics can be applied to many units of analysis: a random collection of people; a family; an economic class or racial group; a neighborhood, city, region, or country; a commercial enterprise; or a subpopulation in any of these populations. Data must be available for the unit of analysis.

Although we can envision models (and metrics) that account for the range of political, social, and economic constructs thought necessary to improve population health, we must decide whether metrics should be selected for all constructs — or whether it is even politically possible to apply incentives across a broad range of areas. American culture is highly pluralistic and politically resistant to such a large-scale, comprehensive approach. No single body controls all these aspects of American public, private, personal, and organizational life enough to hold accountable all entities to which potential incentives apply.

It may be wiser to choose metrics associated with better health for a specific economic, racial, or ethnic group, for example, than for all groups collectively. Even this narrower focus on one group's health can be politically challenging if it is seen to be at the expense of another group or stigmatizes that group.

## The Essays

The essays in this issue of *Preventing Chronic Disease*, solicited on behalf of the MATCH project, describe the characteristics of metrics and provide advice, support, and caution regarding their selection. They characterize the ideal metrics as having the following characteristics:

- simple, sensitive, robust, credible, impartial, actionable, and reflective of community values (1)
- valid and reliable, easily understood, and accepted by those using them and being measured by them
- useful over time and for specific geographic, membership, or demographically defined populations (2)
- verifiable independently from the entity being measured
- politically acceptable
- sensitive to change in response to factors that may influence population health during the time that inducement is offered
- sensitive to the level and distribution of health in a population (2,3)
- responsive to demands for evidence of population health improvement by measuring large sample sizes (4)

Metrics associated with structure (inputs or activities in the logic model framework, eg, the number of people employed or the number of people who have received training in some aspect of their work) or process (eg, the number of activities undertaken in the service of an outcome) could be considered if theory or practice associates these metrics with population health or precursors to population health. "Outcome" metrics measuring specific aspects of clinical health or the cultural foundations that influence clinical health may be desirable, but change in the outcome(s) of interest may not be achievable soon enough for reasonable incentives to be applied.

Measures of people (demographics), the things they do (behaviors), the things that are "done" to them (policy and practice), or their context may be of interest (5,6). A model that recognizes interconnectedness argues for one or more metrics for each of these domains of influence and may reward the type of collaboration and accountability necessary for sustained improvement. Metrics associated with collaboration and accountability can be selected. Increasing evidence indicates that social and economic environments shape resources, opportunities, and exposures, which themselves are outcomes subject to influence and, therefore, rich as a source of metrics (6).

Measurable health outcomes are not just influenced directly (6). For example, health outcomes are subject to changes in crime, environmental hazards, or socially patterned sources of toxic exposures such as landfills, power-generating facilities, truck idling lots, or congested roadways near neighborhoods. Changes in such place-based attributes may be measured in the short term as ends in themselves or as associated in the longer term with measurable clinical outcomes. Aspects of neighborhood (crime, poverty, social distrust, and discrimination) are stressors that can lead to disease through direct neural, neuroendocrine, and immune system pathways. Other indirect pathways include access to housing, food, health care services, or employment opportunities, which themselves are measurable.

Individual or composite metrics can be selected (3). Individual metrics measure a single factor (one contributing to an outcome, eg, the number of people receiving a particular service or benefit) or an outcome itself (eg, numbers or rates of obese people). Composite metrics combine many individual metrics into an aggregate metric thought to better represent the totality of effort. Rankings of the best colleges or communities often reflect this approach. Composite metrics add an element of subjectivity because they ultimately depend on how each component in the aggregate is weighted. These weightings present a political challenge. The entities being offered incentives should concur that the weightings are realistic or relevant.

A successful population-based health incentive system will use metrics that account for the object of the incentives, that can identify change in the timeframe during which the incentives are available, that are realistic for the resources in hand to effect the change desired, and that can be measured effectively (7). We can choose metrics on the basis of what is known to work or allow experimentation. Quality improvement culture demands experimentation, but on the other hand, using proven metrics can force standardization of process before that practice is known. Metrics that ignore countervailing conditions, insufficient time, or political obstinacy can lead to inappropriate reward or penalty.

Lasting interventions that affect population health occur at multiple levels: upstream with large population effect (eg, regulation, taxation, access, economic incentives), midstream (eg, worksite programs), and downstream (eg, individual approaches) (3). Ideally, metrics would be chosen to reflect each of these levels. Such a metric-based performance improvement process would encourage cross-sector collaboration and recognize the systemic precursors to population health.

## Summary

The following guidelines can help ensure that metrics are applied in meaningful ways for rewarding improved population health:

- Determine the problem that needs to be solved.
- Create a visual model that explains the causes of the problem and potential solutions.
- Use an acceptable metric to measure the problem over time so that change can be objectively documented.
- Approach selection of the problem, the solutions to be attempted, and the methods associated with each keeping continuous quality improvement in mind.
- Use a metric that can quantify the problem in real time at the beginning and end of the incentive period.
- Choose a characteristic to measure that is amenable to change.
- Choose a reward or penalty associated with the metric that is of sufficient value to induce the intended change.
- Ensure that the entity being offered the incentive has sufficient control over itself and others to change in ways and magnitudes measureable by the metric.
- Ensure that the entity has sufficient resources (eg, staff, funding, influence, authority) to effect the change.
- Determine when the incentive will be awarded (eg, at the start of the effort to effect change, throughout the effort to produce change, or withheld pending final measurement).
- Assure that the incentive associated with the metric will be awarded.
- Plan to develop new metrics if present metrics prove inadequate.

The challenges associated with choosing the right metrics are many and in some sense antithetical to the ways American political, social, and economic systems work. We often chafe under regulatory and financial frameworks and game such systems to our own advantage. We can be oriented to self rather than to the "public." Nevertheless, it is possible over time to build the broader consensus necessary to improve population health. After all, as a society, we have reduced exposure to tobacco, built sanitary sewer and water systems, achieved nearly universal childhood vaccination, and met other population health goals that were once considered unlikely. Metrics are the means through which we can continue to help communities see the value of working collaboratively for the health of their residents.
